# Laparoscopic spleen-preserving distal pancreatectomy for a solid-cystic intraabdominal desmoid tumor at a gastro-pancreatic lesion: a case report

**DOI:** 10.1186/s12893-020-0691-5

**Published:** 2020-02-03

**Authors:** Keishi Sugimachi, Tomohiro Iguchi, Mitsuhiko Ohta, Yohei Mano, Terumasa Hisano, Ryohei Yokoyama, Kenichi Taguchi, Masahiko Ikebe, Masaru Morita, Yasushi Toh

**Affiliations:** 1 Department of Hepatobiliary-Pancreatic Surgery, National Hospital Organization Kyushu Cancer Center, 3-1-1 Notame, Minami-ku, Fukuoka, 811-1395 Japan; 2 Department of Gastroenterological Surgery, National Hospital Organization Kyushu Cancer Center, Fukuoka, Japan; 3 Department of Hepato-Biliary-Pancreatology, National Hospital Organization Kyushu Cancer Center, Fukuoka, Japan; 4 Department of Orthopedic Surgery, National Hospital Organization Kyushu Cancer Center, Fukuoka, Japan; 5 Department of Pathology, National Hospital Organization Kyushu Cancer Center, Fukuoka, Japan

**Keywords:** Desmoid tumor, Cyst, Spleen-preserving distal pancreatectomy, Case report

## Abstract

**Background:**

We report a case of an intraabdominal desmoid tumor that occurred at a gastro-pancreatic lesion with spontaneous cystic features, and present the successful laparoscopic resection of the tumor.

**Case presentation:**

A 20-mm retroperitoneal cystic mass with a solid component was found adjacent to the stomach and pancreatic body in a 52-year-old woman with no history of familial adenomatous polyposis. Laparoscopic spleen-preserving distal pancreatectomy with wedge resection of the stomach was performed, and complete resection was achieved without intraoperative and postoperative complications. Histopathological examination by immunohistochemistry enabled diagnosis of a desmoid tumor that had originated from the stomach and invaded the pancreatic parenchyma with a spontaneous cystic change. We herein report an extremely rare case of an intraabdominal desmoid tumor with a spontaneous cystic change.

**Conclusion:**

Regardless of its rarity, desmoid tumor should be included in the preoperative differential diagnosis of a cystic intraabdominal mass, and laparoscopic function-preserving surgery may be an optimal treatment option.

## Background

Desmoid tumors (also called aggressive fibromatosis, deep musculoaponeurotic fibromatosis) are locally aggressive tumors with no potential for metastasis or dedifferentiation. Radiologically, they are usually non-specific, slow-growing solid masses that are difficult to distinguish from other soft tissue tumors, but they rarely show cystic lesions. Sporadic desmoid tumors can occur at any site in the body, and anatomic locations are classified into 3 regions: trunk/extremity, abdominal wall, and intraabdominal. Complete surgical resection is the only curative option, but intraabdominal desmoid tumors are often unresectable, because they often infiltrate the mesentery. Here, we report a case of an intraabdominal desmoid tumor that occurred at a gastro-pancreatic lesion with cystic features, and presents the successful laparoscopic resection of the tumor.

## Case presentation

**Fig. 1:**
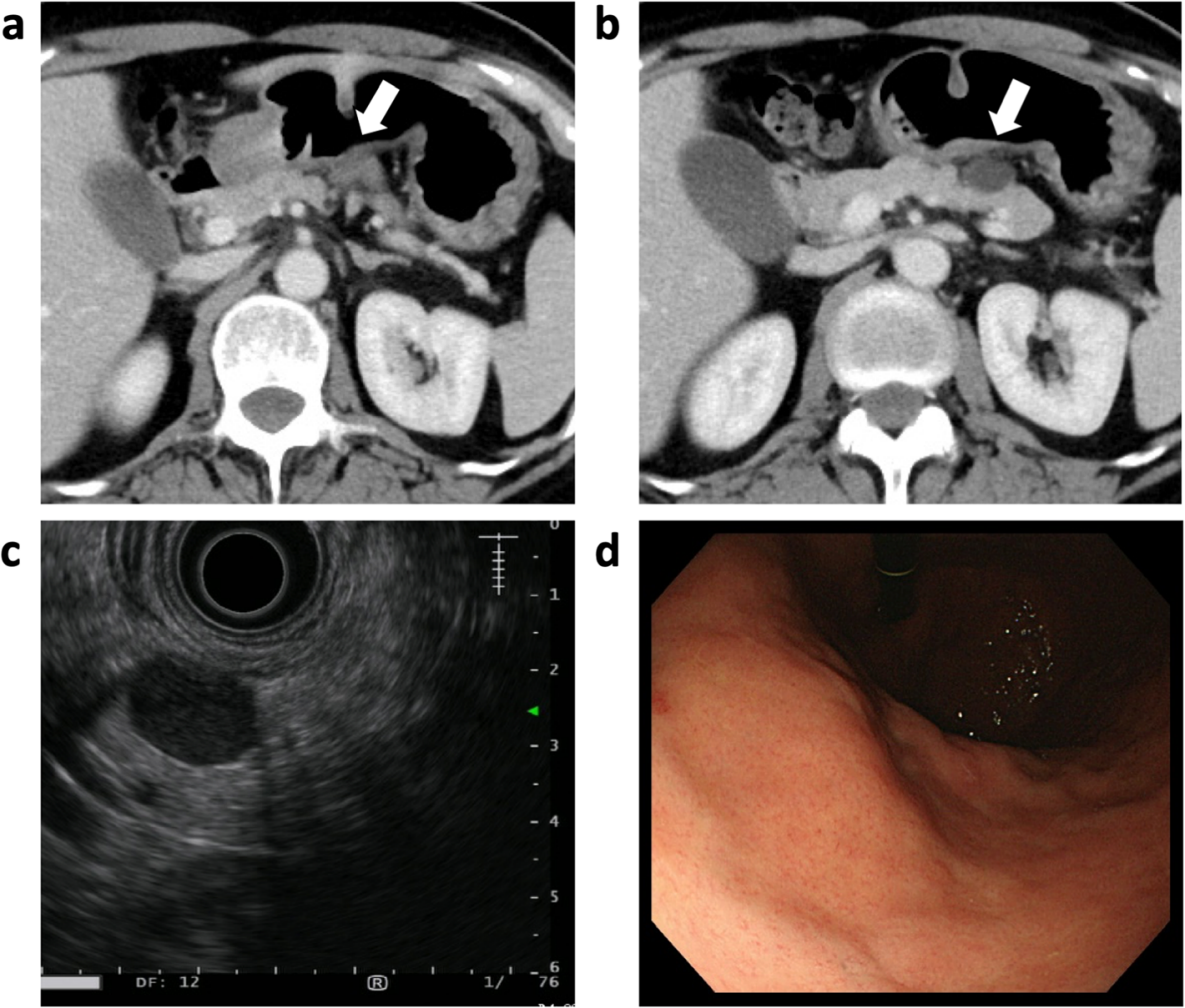
Preoperative images. **a, b** CT image showing a well-defined round, 20-mm cystic mass with a rim of soft tissue in the gastro-pancreatic region. The tumor is dense, adjacent to the gastric wall and pancreatic parenchyma. Slight enhancement of the solid component is seen after intravenous administration of contrast material. Arrows indicate the tumor. **c** Endoscopic ultrasound image showing a well-defined cystic mass. **d** Gastric endoscopy showed no abnormal findings of the gastric mucosa

An intraabdominal cystic mass was incidentally detected between the stomach and the pancreas in a 52-year-old woman undergoing postoperative follow-up computed tomography (CT) for chondrosarcoma of a rib. Her family history was unremarkable. Blood counts and serum biochemistry results were within normal limits. An abdominal CT scan revealed a 20 × 18-mm well-defined cystic mass with a solid component (Fig. 1a,b). The tumor was adjacent to the stomach and the pancreas body, but there was no sign of invasive growth to the organs. MRI showed a cystic mass with high intensity on T2-weighted images and low intensity on T1-weighted images. The lesion showed no sign of diffusion restriction by diffusion-weighed MRI. The solid component of the cystic wall was gradually enhanced in the late phase. Subsequent endoscopic ultrasonography (EUS) showed an 18 × 13 mm round cystic mass, with a heterogeneous nodular lesion at the side of the serosa of the stomach (Fig. 1c). Esophago-gastric endoscopy showed no local lesion on the mucosal surface of the stomach (Fig. 1d). An intraabdominal tumor was suspected, but it was difficult to determine the specific diagnosis.

**Fig. 2:**
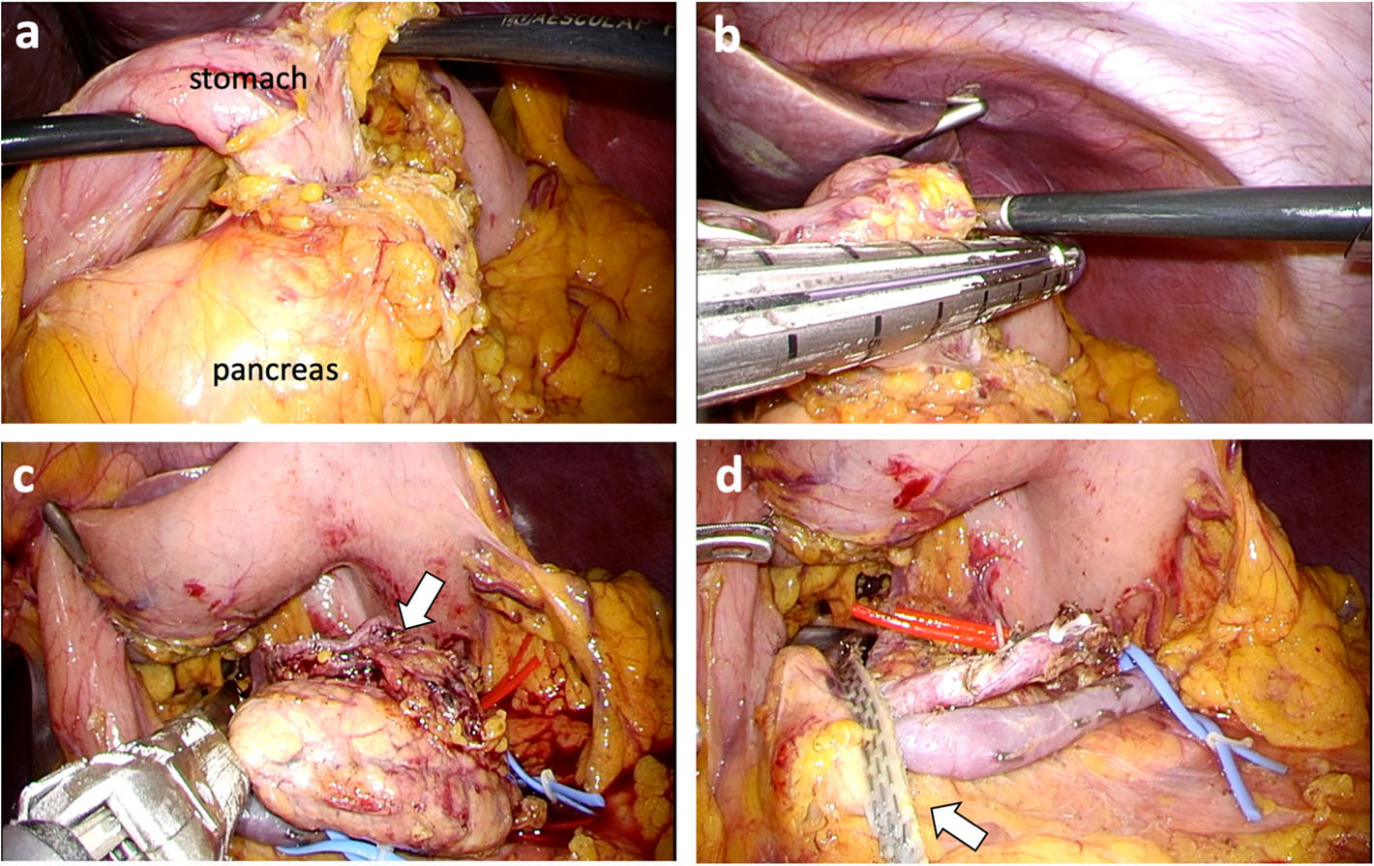
Intraoperative findings**. a** The tumor was located in the gastro-pancreatic region and locally invaded the gastric wall and the pancreatic parenchyma. The arrow indicates the tumor. **b** Wedge resection of the stomach was performed using a linear stapler. **c** The pancreatic parenchyma was transected using a linear stapler. The arrow indicates the tumor. **d** The splenic artery (red tape) and splenic vein (blue tape) were preserved. The arrow indicates pancreatic stump

Laparoscopic resection via 5 trocars was performed with the patient in the supine position. After the gastro-colic ligament was cut, the omental bursa was opened widely. The tumor was confirmed at the superior border of the pancreas body, and adhered to the posterior wall of the stomach (Fig. 2a). With careful dissection, the left gastric artery and vein were isolated and preserved. The tumor was cut from the stomach by wedge resection of the gastric wall using a linear stapler (Fig. 2b). Then, we performed a spleen-preserving distal pancreatectomy. The pancreas body-tail was carefully mobilized and separated from the splenic artery and vein by dissecting the small vessels. The pancreas was dissected at the right side of the tumor using a linear stapler (Fig. 2c). We performed a complete laparoscopic excision, and the spleen and splenic vessels were completely preserved (Fig. 2d). The operative time was 279 min, and intraoperative blood loss was 15 mL. The postoperative course was uneventful, and the patient was discharged healthy.

**Fig. 3:**
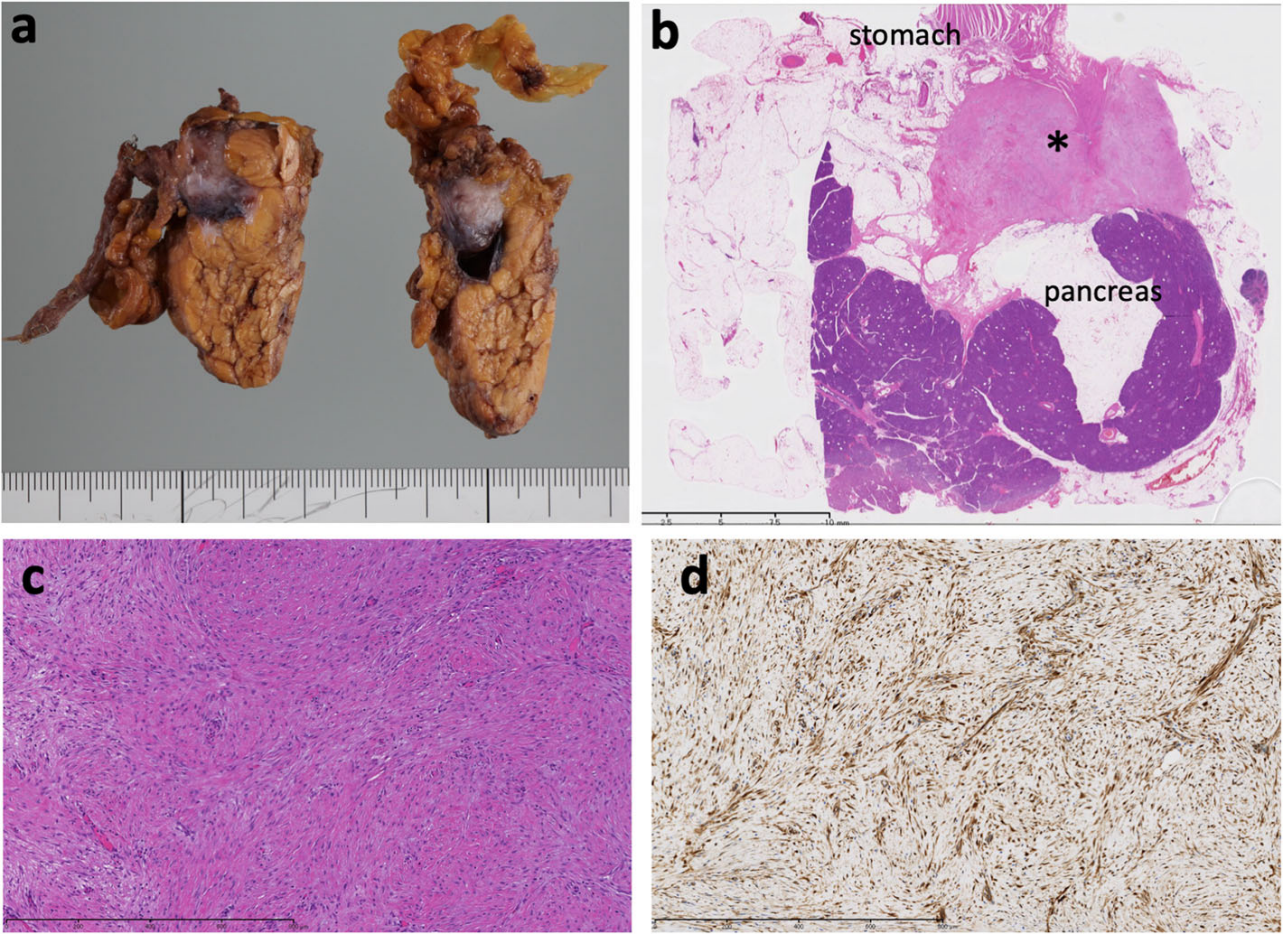
Macroscopic and microscopic findings. **a** The cut surface of the excised solid-cystic mass showing light tan glistening tissue. The arrow indicates the tumor. **b** A lower power view shows that the tumor cells (asterisk) originated from the gastric wall and attached to the pancreatic parenchyma without invasion (hematoxylin and eosin staining; original magnification × 40). **c** Histopathological findings show uniform cord-like proliferation of spindle-shaped cells with minimal atypia, and the intercellular spaces are filled with thick bundles of collagen fibers (hematoxylin and eosin staining; original magnification × 100). **d** Immunohistochemically, the spindle cells are diffusely and strongly positive for nuclear β-catenin protein. (× 100)

Macroscopic examination of the resected specimen revealed a capsulated cystic mass measuring approximately 20 mm with serous content. The whitish solid nodule containing the cystic lesion existed at the wall of the stomach (Fig. 3a). Microscopic examination demonstrated the distribution of spindle tumor cells, partly arranged in a fascicular fashion and accompanied by a collagenous stroma (Fig. 3b, c). Mitotic figures were rarely seen. The tumor cells were attached to the muscular layer of the gastric wall (Fig. 3b). A benign cystic lesion was also observed, but its origin was not determined. Immunohistochemically, the spindle cells were diffusely positive for β-catenin protein in the nucleus (Fig. 3d), but negative for theα-SMA, desmin, S-100, c-kit, and CD34 proteins. These findings were consistent with the diagnosis of intraabdominal desmoid tumor.

## Discussion and conclusions

Desmoid tumors are rare, and their estimated incidence in the general population is 2–4 per million per year [1]. Individuals between the ages of 15 and 60 are most commonly affected, and most desmoid tumors arise sporadically; however, between 5 and 15% are associated with familial adenomatous polyposis (FAP, Gardner syndrome) caused by mutations in the *APC (adenomatous polyposis coli)* gene [2]. The risk of developing a desmoid tumor in a patient with FAP is 852 times that of the general population [2]. Intraabdominal desmoid tumors are usually asymptomatic and therefore are often found incidentally. Furthermore, lack of typical imaging features makes preoperative diagnosis of desmoid tumors very difficult. Typically, imaging studies show a well-defined solid mass with homogenous enhancement and absence of intraabdominal necrosis or degeneration, which reflects the highly collagenous stroma of the tumor [3].

Desmoid tumors potentially show cystic changes with medical treatment or abscess formation [4], but a spontaneous cystic change, as described in this case, is extremely rare, with only eight cases reported in the literature (Table 1) [5–11]. Spontaneous cystic changes could be caused by regression of the tumor associated with the withdrawal of estrogenic stimulation, infection or secondary infarction [11]. In the literature, four pancreatic solid-cystic desmoid tumors have been reported, although pancreatic desmoid tumors are extremely rare (Table 1) [6, 8–10]. In this case, the tumor originated in the stomach and infiltrated the adjacent pancreatic body as confirmed pathologically. It is possible that desmoid tumors at pancreatic lesions are more frequently related to cystic changes; however, further accumulation of cases is warranted to explain this cystic change.

**Table 1:** Reported 8 cases of spontaneous cystic desmoid tumor

No	Primary site	First author	Year	Journal	Reference
1	Mesentery (2 cases)	Ko, SF	2006	Ultrasound Med Biol	[5]
2	Pancreas	Amiot, A	2008	JOP	[6]
3	Mesentery	Tan, CH	2010	Br J Radiol	[7]
4	Pancreas	Xu, B	2013	World J Gastroenterol	[8]
5	Pancreas	Mourra, N	2015	Gastroenterology	[9]
6	Pancreas	Patel, HD	2017	ACG Case Rep J	[10]
7	Retroperitoneum	Lee, KC	2018	BMC Med Imaging	[11]

The mesentery and retroperitoneum are common anatomic locations of intraabdominal desmoid tumors. In the current case, the tumor originated from the stomach, and locally invaded the retroperitoneum and pancreas body, based on the histopathology of the resected specimen. Desmoid tumors can theoretically arise from any part of the body, but those arising from the stomach are extremely rare [12]; thus, mortality is unknown. Desmoid tumors of the remnant stomach may be associated with surgical trauma from a previous gastrectomy [13]. To the best of our knowledge, this is the first report of a desmoid tumor of the stomach with cystic change that originated in a patient with no history of FAP or surgical trauma.

Intraabdominal desmoid tumors rarely metastasize, but may show local invasion and recurrence [14]. The recurrence rate for desmoid tumors is reported to be 30–40%, but that of sporadic desmoid tumors is lower than in patients with FAP [14]. Therefore, complete surgical resection with negative margins is required to achieve curability. We performed a laparoscopic spleen-preserving distal pancreatectomy (SPDP) combined resection of the gastric wall. Considering the oncologic prognosis of desmoid tumor, the surgical margin must be secured to achieve complete resection, but neither lymph node dissection nor wide resection were needed. We chose distal pancreatectomy to avoid the risk of histologically incomplete resection by the extirpation of the tumor. SPDP is beneficial for patients, because it can prevent an increased risk of overwhelming post-splenectomy infection by preserving the splenic immunological function [15]. Laparoscopic surgery is desirable not only because it is minimally invasive, but also because it favors spleen preservation due to a magnified laparoscopic view, which allows for safe dissection of the small pancreatic vessels [16].

In conclusion, we encountered an extremely rare cystic desmoid tumor at a gastro-pancreatic retroperitoneal site that was difficult to diagnose preoperatively. Laparoscopic SPDP with wedge resection of the stomach was performed safely; such procedures could offer an optimal method to achieve both curability and preservation of function.

## Data Availability

Not applicable.

## Abbreviations

CT: Computed tomography
EUS: Endoscopic ultrasonography
FAP: Familial adenomatous polyposis
SPDP: Spleen-preserving distal pancreatectomy
